# Exploring community perspectives on autism genetics research: Indications of supportive views and educational needs

**DOI:** 10.1177/13623613251384342

**Published:** 2025-11-12

**Authors:** Melanie M de Wit, Janneke R Zinkstok, Riley Buijsman, Abdel Abdellaoui, Sander Begeer, Tinca JC Polderman

**Affiliations:** 1 Vrije Universiteit Amsterdam, The Netherlands; 2 Radboud University Medical Centre, The Netherlands; 3 Karakter Child and Adolescent Psychiatry, The Netherlands; 4 Amsterdam University Medical Centre, The Netherlands

**Keywords:** Behavioural genetics, community perspectives, genetic testing and counselling, genetics, stakeholders

## Abstract

Autistic advocates have raised substantial concerns regarding genetics research on autism. It is yet unclear whether these concerns reflect the perspectives of the broader autism community. In a sample of 1757 Dutch autistic adults, 445 parents of autistic children and 126 legal representatives of autistic adults, we explored perspectives on genetics research. We used a pilot repeated survey, consisting of questions about the perceived importance of the heritability of autism, reasons for this importance and the desire to learn more about heritability. Over 95% of the participants found it at least a little important to know that autism is highly heritable, and 67% would like to learn more about it. Participants mentioned that they find it important to increase knowledge about the causes of autism, and to improve the diagnostic process. There were no substantial differences in perspectives between stakeholder groups. Contrasting previous work, this pilot study indicates support for genetics research in autism, which highlights diversity in community perspectives. It also identifies an unmet educational need. We conclude that education on the topic of genetics, active research involvement of the autism community, and an ongoing dialogue between all parties are crucial to ethically and meaningfully move autism genetics forward.

**Lay abstract:**

Some autistic people have shared strong concerns about research on the genetics of autism. However, this has not been investigated systematically in a large and diverse group of stakeholders. Therefore, researchers asked questions to over 1700 autistic adults in the Netherlands, 445 parents of autistic children and 126 legal guardians of autistic adults. The questions were (a) ‘is it important to know that autism is heritable?’, (b) ‘why is this important to know?’ and (c) ‘would you want to learn more about the heritability of autism?’. Over 95% of the people said it is at least a little important to know that autism is heritable. Around 67% also said they would like to learn more about it. Many people said that learning about the genetics of autism could help us understand the causes of autism better and could help improve the diagnostic process. This study shows that there are different opinions about genetics research. It was also noted that people need clear and simple information about autism genetics. To make autism genetics research better and more respectful, it is important to give clear information about genetics, to involve autistic people and their families in research, and to have open conversations between researchers and the autism community. This way, autism genetics research can move forward in a way that is fair and helpful for everyone.

Autism genetics research is growing rapidly and has consistently shown that genetic factors substantially contribute to autism’s aetiology. Early twin and family studies, both study designs that elucidate the influence of genetic and environmental influences on traits (de Wit & Polderman, 2023), indicated that autism is approximately 64%–91% heritable (Tick et al., 2016). Over the past decade, genetics research has partly shifted its focus from these foundational designs to molecular genetic studies. The latter uncovered that autism’s genetic underpinnings are highly complex and heterogeneous, with both common genetic variants with small individual effects and rare variants with large effects influencing its aetiology.

Among many molecular genetic approaches, genome-wide association studies (GWAS) have been particularly informative. GWAS test for associations between a large number of common genetic variants and one or multiple outcomes, for example, the presence of autism. The latest GWAS of autism, performed by Grove et al. (2019), identified five genetic variants significantly associated with an autism diagnosis. However, their findings also showed that autism is highly polygenic, that is, it is influenced by hundreds of common genetic variants with individually small effects. To capture these small effects, large sample sizes are needed. As a result, in recent years many large-scale studies investigating the genetic architecture of autism have been launched. These studies are typically initiated by neuroscientists and aim to elucidate the genetic mechanisms underlying autism and its interplay with environmental influences, enhancing early detection and developing therapeutic strategies (Hens et al., 2016).

The international autism community has raised concerns about these studies, including concerns about privacy issues, transparency of data use and data sharing (Natri, 2021; Natri et al., 2023), in particular in the last 5 years. In addition, several studies highlight a disbalance between the autistic community’s research priorities and the type of research funded. While the community’s priorities comprise help and real-life implications, a large part of the funding goes to fundamental research, including genetics (Cage et al., 2024; Pellicano et al., 2014; Roche et al., 2021).

Perhaps the most pressing concerns, however, are centred around the identification of genetic variants associated with autism and the fear that this may lead to the misuse of this information in eugenic practices (Ellis & Asbury, 2023; Gallion et al., 2025). Eugenics, as defined by Galton, is ‘the science that deals with all influences that improve the inborn qualities of a race’ (Galton, 1904). Even though human behaviour is aetiologically too complex to reliably predict from genetics alone (Forzano et al., 2022; Leblond et al., 2024; Murray et al., 2021), the potential of practices such as embryo selection cause autistic people to feel extremely stigmatized and fear potential eradication of autism.

Fear is likely also enhanced since much of medical research, and genetics research in particular has a medicalized incentive (e.g. ‘curing autism’), which stands in direct contrast with the growing neurodiversity movement that emphasizes autism as part of natural human variation. In the past years, this idea that autism is not a ‘disorder’ but rather a variety of neurodiversity has gained support, and advocates urge society to be more inclusive of neurodivergent people, reduce stigma and reject the medicalized framing of autism (Pellicano & Stears, 2011).

Although voices expressing concerns about genetics research in autism are strong, it is currently unclear to what degree they reflect the perspectives of the broader autistic (i.e. autistic individuals) and autism community (e.g. parents, legal representatives, researchers and clinicians). Recognizing the importance of incorporating various community perspectives, the field of autism research is gradually putting participatory processes at its cornerstone (Fletcher-Watson et al., 2019; Nicolaidis et al., 2019; Pellicano & den Houting, 2022). It is therefore remarkable that community perspectives on autism genetics research, a topic that has proven to be highly controversial, have only been explored sparsely.

The literature to date has primarily explored parents’ perspectives on autism genetics who generally hold a positive attitude towards diagnostic genetic testing for their children (Asbury et al., 2023; Chen et al., 2013, 2015; Johannessen et al., 2017, 2022; Reiff et al., 2015; Wagner et al., 2020; Xu et al., 2018; M. Zhou et al., 2023). Reasons for this positive attitude are mostly fundamental; they indicate genetic testing may help in finding causal explanations, may help with a better understanding of autism, and may aid in developing better interventions. Some parents have also indicated family planning as a reason to do clinical genetic testing (Chen et al., 2015). In these studies, only a small number of parents expressed concerns about diagnostic genetic testing. These concerns were mostly centred around privacy and insurance issues and their child’s ‘right not to know’.

By contrast, the limited research on autistic adults’ perspectives reveals significant concerns. The first quantitative study on this topic showed that many autistic adults held negative views towards clinical genetic testing for autism (Byres et al., 2023). Specifically, 83% of participants expressed that (clinical) genetic testing could increase discrimination against autistic people, while only 24% believed it could increase societal acceptance. In addition, 49% opposed genetic testing altogether, with 39% believing that it could only be harmful to the individual and the community. An important finding of this study is that 74% of the included participants had concerns about autism research. Participants also had hopes for potential benefits such as earlier diagnosis, increased diagnostic certainty and greater acceptance by family members, but these perceived benefits were seemingly outweighed by potential harms.

Further studies echoed these concerns, albeit with some nuanced differences. For example, a more recent study found that while a still significant proportion of autistic adults (57.8%) feared genetic testing would lead to discrimination, attitudes were somewhat more varied and mildly positive on average (Gallion et al., 2025). Findings from a qualitative study parallelled these quantitative findings and provided deeper insights into perceived concerns and benefits. Based on their participants’ input, the authors concluded that for genetics research to move forward, it needs to be more transparent, value-neutral, neurodiversity-affirming and aligned with community priorities (Ellis & Asbury, 2023).

Three major shortcomings emerge from the literature. First, research exploring community perspectives on autism genetics is highly skewed towards *clinical genetic testing*, aimed at reliably identifying rare genetic, often monogenic causes of autism for diagnostic and intervention purposes on an individual level, rather than *research-focused genetic testing*, aimed at studying group differences, often based on polygenic genetic architectures and intended to answer scientific questions. In some instances, both are studied, yet the distinction is not made clear to the participant (Byres et al., 2023). Second, the available literature is focused mainly on parent perspectives rather than that of autistic individuals. This is concerning, considering that the perspectives and priorities of autistic adults often differ from those of the broader autism community (Hens et al., 2016; Pellicano & Stears, 2011). Third, few standardized questionnaires are available to reliably explore and compare community perspectives on autism genetics. To the best of our knowledge, no standardized questionnaire on the topic of research-focused genetic testing for autism exists.

At the Netherlands Autism Register (NAR), we gauged interest in genetics research prior to the creation of a project involving genetics. The NAR performs longitudinal research to improve the lives of autistic individuals. It centres around participatory research, with autistic people involved in all stages of the research process. A priority-setting project within our cohort, led by two autistic researchers and in close collaboration with multiple Dutch autism organizations, gauged stakeholders’ research priorities. This large-scale inventory, including over 900 NAR participants, identified ‘causes, mechanisms and prevention’ as one of the prominent themes across three different stakeholder groups; autistic adults, parents of autistic children, and legal representatives (*https://onderzoeksagenda-autisme.nl/*). This theme included, but was not limited to, genetics research. The survey that was used in the current study was aimed at exploring this interest further; a brief survey in the yearly NAR questionnaire of 2018 included questions about the perceived importance of the heritability of autism, and whether the NAR participants were interested in more research and information about genetics and autism. Ultimately, after careful consideration of participants’ perspectives, a project on the interplay between environmental and genetic factors was co-created with a team of autistic and non-autistic stakeholders and researchers. The survey was repeated in 2023 to assess whether opinions on genetics research remained unchanged.

Based on this pilot repeated survey, the current study aims to explore the perspectives of autistic adults, parents of autistic children, and legal representatives of autistic adults on autism genetics research. We do so by assessing if and why participants find it important that autism is highly heritable and if they would like to learn more about its heritability. We also study predictors of the perceived importance of autism heritability and the desire to learn more. For this, we focus on (a) age of diagnosis (i.e. during childhood or in adulthood), since this may reflect different presentations of autism, (b) whether a family member has autism, (c) if genetic testing was offered, since both these factors may increase awareness of genetic influences on autism and (d) educational attainment. Finally, we assessed whether perspectives on genetics research have changed over a 5-year period.

## Inclusive language statement

In line with previously reported preferences of our participants (Buijsman et al., 2023), throughout this report, we use both identity-first (autistic person) and person-first (person with autism) language.

## Methods

### Participants

Participants were a total of 2328 members of the NAR that completed the yearly questionnaire and included three different stakeholder groups: 1757 adults (16+) with autism, 445 parents of autistic children (16−) and 126 legal representatives of autistic adults (16+) with high support needs. Participants can voluntarily register for the Netherlands Autism Register through the website (https://nar.vu.nl/#), after which they receive a first questionnaire consisting of items on demographics, personal characteristics, family dynamics and living situation, the diagnostic process, co-occurring diagnoses, interventions and medication use, daily activities and general wellbeing. After filling in this baseline questionnaire they are invited to participate in a yearly questionnaire of variable content. All questionnaires are distributed through email and are in Dutch. Autistic participants received a formal autism diagnosis from a qualified clinician according to the Diagnostic and Statistical Manual of Mental Disorders (DSM). Parents and legal representatives reported for their child/person they represented. The legal representatives are a diverse group of individuals including mostly family members of autistic adults requiring (very) substantial levels of support. The majority of NAR participants identify as Dutch (>95%).

**Figure 1.**
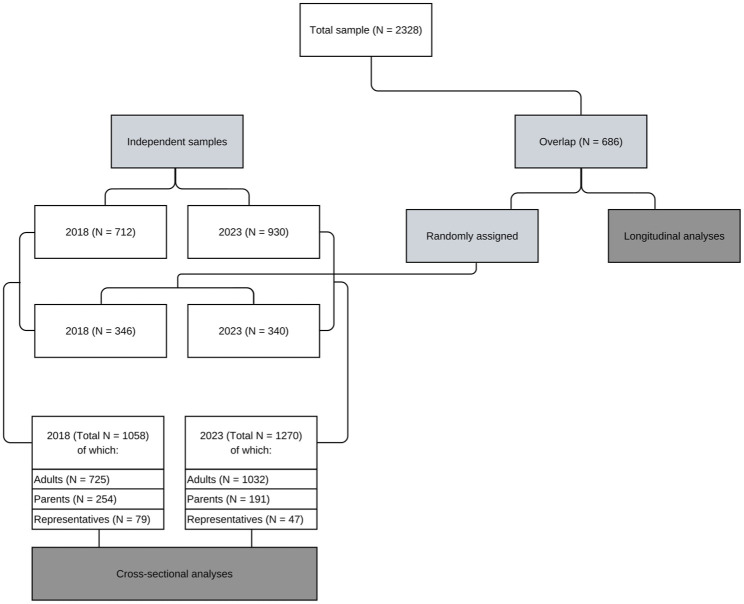
Participant inclusion and allocation to waves.

A total of 686 participants filled in both the 2018 and 2023 questionnaires. These participants were randomly allocated to either one of the questionnaires to ensure independent samples for subsequent cross-sectional analyses. A flowchart of participant inclusion and allocation to these waves is presented in Figure 1.

The Netherlands Autism Register received ethical approval for their research from the ethical committee of Vrije Universiteit Amsterdam (VCWE 2020-041R1), and the genetic project specifically from the Amsterdam UMC Medical Ethical Committee (NL79422.029.21). All included individuals provided written informed consent.

### Materials

Participant demographics were collected online at the baseline questionnaire and the yearly questionnaires in 2018 and 2023. Perspectives on genetics research were collected in the yearly questionnaires in 2018 and 2023. The survey used in this study was specifically designed to gauge the register’s participants’ interest in pursuing a project inclusive of genetics rather than to answer scientific questions. Since existing validated questionnaires did not adequately capture the variables of interest, a new questionnaire was developed by the research team and reviewed by a stakeholder panel (as part of the yearly stakeholder review procedure for the full questionnaire) for relevance and clarity. Based on their advice, one question was removed from the initial survey: ‘What is your view on curing autism (should that become possible)?’. The final survey included items on whether diagnostic genetic testing was offered, the perceived importance of the heritability of autism, reasons why they found this important, knowledge about the heritability of autism and the desire to learn more about it. A detailed description of all included measures is presented in Table 1.

**Table 1.** Description of demographic and genetics-related measures.

Variable	Answer options
Role of participant	Autistic adult, Parent of autistic child, legal representative of autistic person
Age	In years and months
Age at diagnosis	In years and months
Sex assigned at birth	Male, Female
Gender	Man, woman, between man and woman, both man and woman, not man nor woman, other, don’t know (yet)
Highest achieved education	Practical, vocational, theoretical (classification based on Dutch Centraal Bureau Statistiek Standaard Onderwijsindeling 2016| CBS)
‘How heritable do you think autism is?’	Slider between 0 and 100.
‘Upon receiving my autism diagnosis, genetic testing was offered and I was asked to contribute DNA’	Yes, No
‘Research shows autism is a highly heritable trait. I find this . . .’	‘Important’, ‘A little important’, ‘Not important’
‘I find this important because . . .’	‘This could give us general knowledge about the causes of autism’, ‘This helps me accept myself (or my kids)’, ‘This may contribute to better diagnosis of autism’, ‘This may contribute to better insights into co-occurring conditions or traits, now or in the future’, ‘This may contribute to better help and support’, ‘This may influence further family planning’, ‘Other’. Participants could select multiple answers
‘I would like to obtain more information about the meaning of the heritability of autism’	‘Yes, about the heritability of autism in general’, ‘Yes, about the heritability of autism for me (or my children) specifically’, ‘No’
‘How would you like to receive information?’	Open-ended

### Statistical analyses

Our primary aim was to provide descriptive statistics on all genetics-related questionnaire items. In addition, we used chi-square tests to assess the influences of (a) age at diagnosis (<18 vs >18), (b) having a close family member with an autism diagnosis, (c) having had genetic testing offered and (d) educational attainment, on (1) the perceived importance of autism heritability and on (2) whether participants would like to learn more about heritability. The influence of age at diagnosis, having a family member with autism, having had genetic testing offered, and educational attainment was tested in autistic adults only due to restrictions in the sample size and/or data availability. Finally, we assessed whether perspectives changed over a 5-year period by (a) performing cross-sectional analyses over the total sample and the specific stakeholder groups and (b) performing longitudinal analyses using McNemar tests (McNemar, 1947) to assess change in perspectives in participants that filled in questionnaires during both data waves (i.e. 2018 and 2023). All analyses were performed in the Statistical Package of Social Sciences (SPSS) and RStudio (Posit Team, 2024). We applied Bonferroni correction for multiple testing.

### Participatory methods

This study is part of a project that was set up based on all NAR participants’ research priorities (see introduction for explanation of the NAR mission statement and approach). Autistic team members were involved in all stages of the research and are in the writing team of this specific study. Before sending it out to participants, community advice on the questionnaire used in this study was provided by a panel of autistic adults and parents of autistic children as part of the assessment of the yearly questionnaire. Community advice on the interpretation of the results was provided by an independent panel of people with lived experience who are connected to the project long-term and are consulted on its progression every 6 months (*N* = 5, age range 23–64).

## Results

Since results were largely similar for the two separate waves, out of consideration of conciseness, we present the data of 2023 here while data of 2018 can be found in Supplemental sTables 1 and 2. Comparative analyses between individuals that dropped out after 2018 versus those that remained revealed demographic differences between these groups (highest achieved education, χ^2^ = 11.834, *df* = 4, *p* = 0.003; biological sex, χ^2^ = 12.993, *df* = 1, *p* < 0.001; age, *t* = −12.328, *df* = 1391.4, *p* < 0.001; and age at diagnosis, *t* = −7.3913, *df* = 1216, *p* < 0.001), but no significant differences on genetics-related questionnaire items (importance heritability, χ^2^ = 1.797, *df* = 2, *p* = 0.407; wants to know more, χ^2^ = 0.919, *df* = 1, *p* = 0.338), see Supplemental sTable 3.

### Descriptives

Descriptive demographics per stakeholder group are presented in Table 2.

**Table 2.** Descriptive Demographics for the Total Sample and per Stakeholder Group in 2023.

	Adults (*N* *=* 1032)	Parents (*N* *=* 191)*	Representatives (*N* *=* 47)*	Total (*N* *=* 1270)
Age				
Mean (*SD*)	49.14 (13.36)	14.50 (2.40)	32.06 (12.78)	39.74 (17.46)
Range	20.58–77.89	10.79–20.04	20.35–73.62	4.33–78.66
Age at diagnosis				
Mean (*SD*)	36.18 (14.96)	3.97 (1.97)	5.85 (3.01)	31.34 (17.71)
Range	3.25–67.50	1.00–8.75	2.58–14.50	0.67–70.00
Sex assigned at birth				
Male	351 (34.01%)	136 (71.20%)	34 (72.34%)	521 (41.02%)
Female	661 (64.05%)	55 (28.80%)	13 (27.66%)	729 (57.40%)
Other	20 (1.94%)	0	0	20 (1.57%)
Gender				
Man	323 (34.01%)	132 (69.11%)	-	455 (37.20%)
Woman	557 (53.97%)	51 (26.70%)	-	608 (49.71%)
Between man and woman	50 (4.84%)	5 (2.62%)	-	55 (4.50%)
Both man and woman	13 (1.26%)	4 (2.09%)	-	15 (1.23%)
Not man nor woman	42 (4.07%)	0	-	42 (3.43%)
Other	29 (2.81%)	0	-	29 (2.37%)
Don’t know (yet)	18 (1.74%)	1 (0.52%)	-	19 (1.55%)
Highest finished education				
No education	4 (0.39%)	-	6 (12.77%)	10 (0.93%)
Practical	217 (21.03%)	-	28 (59.57%)	245 (22.71%)
Vocational	160 (15.50%)	-	1 (2.13%)	161 (14.92%)
Theoretical	627 (60.76%)	-	3 (6.38%)	630 (58.39%)
Other	24 (2.33%)	-	9 (19.15%)	33 (3.06%)

* Descriptives are provided for the person that parents and stakeholders represented.

### Cross-sectional analyses

Results on the perspectives on genetics research per stakeholder group are presented in Table 3. In summary, we found that 94.72% of the complete sample find it at least a little important to know that autism is highly heritable. The main reasons listed are ‘to be able to increase knowledge about the causes of autism’ and ‘to allow for better diagnostic assessment of autism’. Two-thirds of participants (67.17%) would like to learn more about the heritability of autism. The most reported means through which they would like to learn more were online (through informative websites) (48.59%), personal conversation (with a genetic counsellor, GP, psychiatrist) (19.49%), through folders, magazines or newspapers (20.03%) and through an autism organization (20.51%). We also found that 5.83% were offered genetic testing. Participants on average estimated the heritability of autism at 75.36% (*SD* = 19.21).

**Table 3.** Descriptive Statistics for Genetics-Related Survey Items for the Total Sample and per Stakeholder Group in 2023.

	Adults (*N* = 1032)	Parents (*N* = 191)	Representatives (*N* = 47)	Total (*N* = 1270)
‘Heritability important fact’				
Important	781 (75.68%)	142 (74.35%)	35 (74.47%)	958 (75.43%)
A little important	189 (18.31%)	45 (23.56%)	11 (23.40%)	245 (19.29%)
Not important	62 (6.00%)	4 (2.09%)	1 (2.13%)	67 (5.28%)
Reasons*				
Knowledge	681 (65.99%)	132 (69.11%)	25 (53.19%)	838 (65.98%)
Acceptance	518 (50.19%)	70 (36.65%)	12 (25.53%)	600 (47.24%)
Diagnosis	700 (67.83%)	115 (60.21%)	27 (68.69%)	842 (66.30%)
Co-occurring conditions	502 (48.64)	96 (50.26%)	25 (53.19%)	623 (49.06%)
Help	175 (16.96%)	32 (16.75%)	9 (19.15%)	216 (17.01%)
Family planning	63 (6.10%)	16 (8.38%)	7 (14.89%)	86 (6.77%)
‘Want to know more about heritability’*				
Yes, in general	581 (56.30%)	90 (47.12%)	23 (48.94%)	694 (54.65%)
Yes, for my family specifically	388 (37.60%)	91 (47.64%)	24 (51.06)	503 (39.61%)
No	346 (33.53%)	60 (31.41%)	11 (23.40%)	417 (32.83%)
Genetic testing offered	42 (4.07%)	19 (9.95%)	13 (27.66%)	74 (5.83%)
Mean estimated heritability (*SD*)	76.45 (18.08)	71.34 (21.88)	67.83 (26.90)	75.36 (19.21)

* It was possible to choose more than one reason.

In a cross-sectional analysis of participants from all three stakeholder groups, participants in 2023 were older (*t* = −5.48, *df* = 2218.4, *p* < 0.001), more often female (χ^2^ = 34.58, *df* = 2, *p* < 0.001) and higher educated (χ^2^ = 106.81, *df* = 4, *p* < 0.001). There were no differences on most genetics-related items (perceived importance of autism heritability (χ^2^ = 3.71 *df* = 2, *p* = 0.157), whether they wanted to learn more about the heritability of autism (χ^2^ = 0.00, *df* = 1, *p* = 1), and whether genetic testing was offered (χ^2^ = 1.68, *df* = 1, *p* = 0.195). Participants in 2023 also did not differ from those in 2018 on the reasons they found autism’s heritability important (*p* ⩾ 0.040), except that there was an increase in perceived benefit of acceptance of participants’ or participants children’s autism due to genetics research (χ^2^ = 12.19, *df* = 2, *p* < 0.001). All cross-sectional results, including those for the total sample and the adults, parents and legal representatives separately, are presented in Supplemental sTable 4.

### Longitudinal analyses

Longitudinal analyses in a subset of 686 participants across all three stakeholder groups did not reveal significant differences between 2018 and 2023 for the perceived importance of autism’s heritability (χ^2^ = 2.59, *df* = 3, *p* = 0.459) or the reasons for why they found it important (*p* ⩾ 0.065), but in 2023, fewer participants reported they wanted to learn more about autism heritability (*p* < 0.001). Results of the longitudinal analyses for the total sample and per stakeholder group are presented in Supplemental sTable 5.

### Predictors

Adults who had been offered genetic testing in the past were more likely to want to learn about it. We did not observe any associations between the perceived importance of autism heritability or the desire to learn more about it, and age at diagnosis, having a first-degree family member with an autism diagnosis, or having been offered genetic testing (*p* > 0.156), see Table 4.

**Table 4.** Predictors of the Perceived Importance of Autism Heritability and Desire to Know More in Autistic Adults in the Year 2023.

	Importance heritability			Desire to know more (No vs yes)		
	χ^2^	*df*	*p*	χ^2^	*df*	*p*
Age at diagnosis (<18 vs >18)	.724	2	.696	3.401	1	.065
Family with autism	6.55	2	.038	1.525	1	.217
Genetic testing offered	1.184	2	.553	8.20	1	.004*
Educational attainment	2.634	4	.621	.053	2	.974

* Significant after Bonferroni correction (.05/8).

Community advice on the interpretation of these findings and future directions are summarized in Box 1.

## Box 1. Community advice on the interpretation of the current results and recommendations for future directions

During this project, community advice was received through different channels: (a) a lived-experience review panel assessed the yearly questionnaire including the items included in this study before sending it out and (b) a lived-experience panel involved in this project provided crucial insights into the interpretation of our findings, and future directions during 3-hour, biannual, live meetings. A summary of the community input we received is listed below.

*Interpretation*:

- These findings underscore the importance of including perspectives from different stakeholder groups (e.g. autistic people, family members of autistic people, legal representatives and clinicians) recruited through different channels (e.g. social media, registers and clinics).
- The panel members restated that understanding the genetic underpinnings of autism could be particularly valuable in increasing acceptance of self by reducing self-blame, as well as acceptance from the community.
- The presence of support for genetics research does not equal the absence of concerns.

*Future directions*:

- Questions asking about concerns with respect to genetics research, and reasons why not to participate, should be included in standardized questionnaires.
- Self-blame should be included as one of the reasons to do genetics research. This may be especially relevant to parents of autistic children.
- Questionnaires on this topic should not address what is impossible (e.g. predicting autism diagnoses from polygenic scores) but rather focus on what is possible. This may prevent unfounded fears.
- The distinction between clinical genetic testing and research-focused genetic testing should be clearly outlined in future work.
- Qualitative studies are needed to gain a deeper understanding of these results and to explore potential underlying concerns of the autistic community.

## Discussion

This study explored the perspectives of autistic adults, parents of autistic children, and legal representatives of autistic adults on genetics research. Our results indicate interest in genetics research in the Dutch autistic and broader autism community, while also identifying an educational need. Offering a stage to understudied groups, these findings offer a novel perspective that both contrasts and aligns with previous literature.

Contrasting previous studies highlighting cautious or negative views among autistic adults towards genetics research (Byres et al., 2023; Ellis & Asbury, 2023; Hendry et al., 2025), our findings present a more positive attitude. Our participants expressed interest in the heritability of autism and a desire to learn more about its genetic underpinnings. Particularly interesting is the finding that merely generating knowledge about the causes of autism, a goal that does not have an immediate impact on autistic people’s everyday lives, is an important reason to be interested in genetics research. This could appear to be at odds with previous studies indicating priority for research that has direct practical implications (Cage et al., 2024; Ellis & Asbury, 2023; Pellicano et al., 2014), however, it should be understood that our study was not aimed at priority setting and our results do not imply that the autism community prioritizes genetics research over other research.

Differences in participants’ characteristics may in part explain why our results differ from previous work. For example, compared to Byres et al. (2023), our participants were more often white and male and were all diagnosed by a healthcare professional, whereas 33% of their participants were self-diagnosed. Compared to Ellis and Asbury (2023), our sample differed in terms of age at diagnosis (their participants were all diagnosed in adulthood). In addition, it is important to note that participants in both previous studies were recruited through convenience sampling on social media, which may have resulted in a selection bias towards those vocal about or critical of genetics research (Byres et al., 2023; Hendry et al., 2025; Rødgaard et al., 2022), whereas we recruited through a longitudinal research cohort, which may have resulted in a selection bias towards those more positive towards genetics research, considering that our participants are already actively participating in autism research.

A study that specifically focused on investigating the perspectives of autistic biobank participants and their family members supports this hypothesis. The results suggest general support for genetics research and overall trust in science (Lilley et al., 2023). This approach naturally offers a different angle than studies that recruit participants on social media, as these biobank participants are already actively participating in biological research. While participants acknowledged some of the concerns raised by the autism community, they were mostly supportive of genetics research and showed trust in science. The participants stated that among their reasons for participation were identifying causal mechanisms, improving diagnostic procedures and improving early interventions, which aligns with perspectives and priorities found in our study.

During our literature search and conversations with our lived-experience panel, we observed an important shortcoming in the existing terminology around genetics research, where the term ‘genetic testing’ is often used to describe both clinical, diagnostic genetic testing and genetic testing for research purposes. This may in part be caused by an attempt to simplify concepts in genetics, because of the unfamiliarity among the community. While it is crucial to make genetics research accessible and easy to understand, oversimplification may increase misunderstandings between researchers and the public (Morosoli et al., 2024). Hence, we strongly recommend dissecting the umbrella term ‘genetic testing’ into two distinct concepts with on one end *clinical genetic testing*, conducted in the clinical setting and aimed at reliably identifying rare genetic causes of autism for diagnostic and intervention purposes on an individual level, and on the other end *research-focused genetic testing*, often based on polygenic scores, performed in the academic research setting and currently not suitable for reliably predicting autism diagnosis on an individual level (de Wit et al., 2025). This observation, in addition to the finding that 2/3^rd^ of our participants would like to learn more about heritability, adds to an existing body of literature that indicates an unmet educational need in the general public on the topic of genetics (Chapman et al., 2019; Gerdes et al., 2021; Hanish et al., 2018; Mills Shaw et al., 2008; Wagner et al., 2020).

This unmet educational need is concerning, given that (a) genetics research is one of the most fast-growing scientific fields, (b) it is becoming increasingly important in clinical practice, (c) it is reaching the media more frequently (Morosoli et al., 2024), and d) the rapidly developing possibility and increased uptake of direct-to-consumer genetics (MIT Technology Review, n.d.), which allows the public to access genetic information without direction from a clinician. In addition, it has been shown that limited genetic knowledge may increase the chance of having a negative reaction to genetic results (Peck et al., 2022). For the public, and the autism community in particular, to understand and interpret genetics research and make informed choices regarding participation in clinical genetic testing as well as research-focused genetics research, its genetic literacy needs to increase.

It should be recognized that a substantial body of autism genetics literature exists, including early twin and family studies that showed the heritability of autism, large-scale genome-wide approaches that identified specific variants associated with autism’s aetiology, as well as an incredible amount of in-depth and topic-specific analyses. Yet, genetic science is moving faster than its dissemination to stakeholders; findings of this work have often not been effectively communicated to the autism community or the general public, which reflects a broader failure of the research community to ensure transparent and accessible communication of genetic knowledge (Fletcher-Watson et al., 2021).

Our results may offer some pointers to effectively increase genetic literacy. We identified several means through which the autism community would like to be informed, the most important one being through online resources. Many parents and autistic adults also indicated they would like to learn about genetics through a healthcare provider. Controversially, healthcare providers often feel they have inadequate knowledge to provide this information (Finn et al., 2005; Haga et al., 2019; Mikat-Stevens et al., 2015; Nurnberger et al., 2018; Soda et al., 2021; Y. Z. Zhou et al., 2014). We underscore the importance of properly educating healthcare providers in genetics and support ongoing efforts to integrate genetics into healthcare education (Guttmacher et al., 2007; Nurnberger et al., 2018). Finally, in line with multiple recent calls (Fletcher-Watson et al., 2019; Hens et al., 2016; Kaljusto et al., n.d.; Natri, 2021; Natri et al., 2023), we advise researchers in the field of autism, and genetics researchers in particular, to seriously invest in community involvement. This can be achieved by thoroughly examining the autistic community’s research priorities, by including autistic individuals in the research team (see, for example, Nicolaidis et al. (2019) for a framework), and by implementing participative research approaches. In addition, efforts should be put into creating an ongoing dialogue between autistic people, their close ones, clinicians and researchers, to closely monitor scientific and societal progress and challenges surrounding autism genetics and to interactively address concerns. We emphasize that hearing all community perspectives, especially those of autistic people, is imperative to move the field forward in an ethical and meaningful way.

This study has some limitations. Our survey was primarily designed to gauge interest rather than answer scientific questions. A custom-made, non-validated survey was used to this end. Therefore, this study should be considered a pilot exploration. A specific, major limitation with respect to our survey is that respondents were not explicitly provided with the option to express concerns surrounding genetics research in the questionnaire, other than being able to email the researcher afterwards. This is important to note since the participants’ interest in genetics research does not necessarily imply the absence of concerns. Previous research on autistic perspectives on genetic testing has shown that although autistic people recognize potential benefits, they tend to be equally or even more concerned about it (Hendry et al., 2025; Klitzman et al., 2024). Similar tendencies could be true for genetics research in the research setting. It is noted that the current study is meant as a first exploration, and that the presented stakeholder input is meant to guide future research aimed at understanding perspectives and concerns around genetics research in the Dutch community better.

Another limitation is that the survey items may have been prone to interpretation bias. For example, we asked participants to estimate the heritability of autism, but did not inform them about the definition of heritability in genetics research. A common misconception about genetics is that *heritability* (the proportion of variance within a population that is explained by genetic differences between people) is confused with *hereditary* (the process of transmitting characteristics from the ancestors to the next generations) (Moore & Shenk, 2017). In addition, our survey did not explicitly outline the difference between clinical genetic testing and research-focused genetic testing. Recognizing that genetic literacy in the general population is low this may have caused incorrect interpretation of the questionnaire items. As demonstrated by Ellis and Asbury (2023) and as indicated by the lived experience panel involved in this project, additional qualitative research may help to obtain a deeper understanding of the answers that were provided in this study. Furthermore, although substantial effort was made to ensure the objectivity of the research design and interpretation of results, it is important to acknowledge that this study was executed by a team of researchers in autism and genetics, psychiatrists, and people with lived experience, whose prior perspectives and experiences may have shaped the design process of the survey and the interpretation of its results. Recognizing these limitations is crucial for putting this study’s findings into context. Hence, it would be of great value to co-create a standardized, validated survey that addresses knowledge, interest, and concerns in the community in order to mitigate these limitations.

Although our research sample is large and represents three different stakeholder groups, participants enrolled in the NAR do not represent the whole autistic community or the broader autism community. First, because participants had already expressed interest in autism research by voluntarily participating in the NAR. Second, participants are on average higher educated than the Dutch population. Finally, by employing an online survey we only included individuals who are able to access the Internet and complete a questionnaire. Another limitation applies to the legal representatives, as this comprises a highly heterogeneous group of parents of autistic adults, other family members, and legal guardians. Hence, these results should be interpreted with caution. Yet this is also a strength, since to the best of our knowledge we are the first to explore views on genetics research in this stakeholder group. Finally, our participants were all located in the Netherlands, which reduces its generalizability to the international community. Due to our sample characteristics, it is important to note that the support we find in this study may not represent the perspectives of the broader autism community, especially those that are sceptical. Rather, our findings add to an incomplete picture of a wide variety of community perspectives.

Despite its limitations, this pilot study offers a stage to understudied groups within the autism community regarding their perspectives on genetics research. Our results offer important and timely insights into the diversity of perspectives and knowledge around genetics research and highlight the importance of including a variety of community voices when conducting genetics research on autism. Crucially, this study is not a call for more genetics research, but rather a call to move the field forward in an ethical way. To do so, it is imperative to invest in meaningfully involving the autistic and autism community in research, to increase genetic literacy, and to actively pursue an ongoing dialogue between stakeholders.

## Supplemental Material

sj-docx-1-aut-10.1177_13623613251384342 – Supplemental material for Exploring community perspectives on autism genetics research: Indications of supportive views and educational needs Supplemental material, sj-docx-1-aut-10.1177_13623613251384342 for Exploring community perspectives on autism genetics research: Indications of supportive views and educational needs by Melanie M de Wit, Janneke R Zinkstok, Riley Buijsman, Abdel Abdellaoui, Sander Begeer and Tinca JC Polderman in Autism
