# Supplementary material for: Exploring community perspectives on autism genetics research: Indications of supportive views and educational needs
Source: Autism. 2025 Nov 12;30(2):390–401. doi: 10.1177/13623613251384342 (PMC12804430; doi:10.1177/13623613251384342)
Supplement: sj-docx-1-aut-10.1177_13623613251384342 – Supplemental material for Exploring community perspectives on autism genetics research: Indications of supportive views and educational needs [file sj-docx-1-aut-10.1177_13623613251384342.docx]

Supplementary materials to ‘**Exploring Community Perspectives on Autism Genetics Research: Indications of Supportive Views and Educational Needs’**

M.M. de Wit, J.R. Zinkstok, R. Buijsman, A. Abdellaoui, S. Begeer, T.J. Polderman

sTable 1. Descriptive Demographics for the Total Sample and per Stakeholder Group in 2018.

|  | Adults (*N =* 725) | Parents (*N =* 254)* | Representatives (*N =* 79)* | Total (*N =* 1058) |
| --- | --- | --- | --- | --- |
| Age |  |  |  |  |
| Mean (*SD*) | 44.61 (13.63) | 12.54 (2.78) | 27.79 (9.89) | 35.68 (18.01) |
| Range | 16.28 – 84.18 | 3.61 – 16.17 | 16.45 – 58.62 | 3.61 – 84.18 |
| Age at diagnosis |  |  |  |  |
| Mean (*SD*) | 36.36 (14.96) | 5.54 (4.72) | 7.22 (5.75) | 28.43 (18.73) |
| Range | 3.08 – 75.50 | 1.50 – 60.00 | 1.67 – 35.50 | 1.50 – 75.50 |
| Sex assigned at birth |  |  |  |  |
| Male | 307 (42.34%) | 198 (77.95%) | 54 (68.35%) | 559 (52.84%) |
| Female | 411 (56.69%) | 56 (22.05%) | 25 (31.65%) | 492 (46.50%) |
| Other | 7 (0.97%) | - | - | 7 (0.66%) |
| Gender** |  |  |  |  |
| Man | 155 | 17 |  | 172 |
| Woman | 187 | 6 |  | 193 |
| Partly man, partly woman | 20 | 0 |  | 20 |
| Not man nor woman | 25 | 0 |  | 25 |
| I don’t know (yet) | 2 | 0 |  | 2 |
| Gender fluid | 1 | 0 |  | 1 |
| Other | 5 | 2 |  | 7 |
| I’d rather not say | 0 | 0 |  | 0 |
| Highest finished education** |  |  |  |  |
| No education | 3 |  |  | 3 |
| Low | 67 | 1 | 28 | 96 |
| Middle | 212 |  | 6 | 218 |
| High | 282 |  |  | 282 |
| Other | 1 | 5 | 2 | 4 |

*Descriptives are provided for the person that parents and stakeholders represented.
**Gender and highest achieved education was assessed differently than in 2023 and were not mandatory items, which resulted in incomplete information for this year. For this reason we deem it irrelevant to present percentages.

sTable 2. Descriptive Statistics for Genetics-Related Survey Items for the Total Sample and per Stakeholder Group in 2018.

|  | | Adults (*N =* 725) | Parents (*N =* 254) | Representatives (*N =* 79) | Total (*N =* 1058) |
| --- | --- | --- | --- | --- | --- |
| ‘Heritability important fact’ |  |  |  |  |  |
| Important | 569 (78.48%) | 200 (78.74%) | 56 (70.89%) | 825 (77.98%) |  |
| A little important | 115 (15.86%) | 40 (15.75%) | 17 (21.52%) | 172 (16.26%) |  |
| Not important | 41 (5.66%) | 14 (5.52%) | 6 (7.59%) | 61 (5.77%) |  |
| Reasons* |  |  |  |  |  |
| Knowledge |  |  |  |  |  |
| Acceptance |  |  |  |  |  |
| Better diagnosis | 474 (69.30%) | 140 (58.33%) | 50 (68.49%) | 664 (66.60%) |  |
| Co-occurring conditions | 350 (51.17%) | 118 (49.17%) | 39 (53.42%) | 507 (50.85%) |  |
| Better help | 406 (59.36%) | 139 (57.92%) | 48 (65.75%) | 593 (59.48%) |  |
| Family planning | 52 (7.60%) | 29 (12.08%) | 6 (8.22%) | 87 (8.73%) |  |
| ‘Want to know more about heritability’* |  |  |  |  |  |
| Yes, in general | 393 (54.21%) | 110 (43.31%) | 33 (41.77%) | 536 (50.66%) |  |
| Yes, for my children specifically | 268 (36.97%) | 120 (47.24%) | 28 (35.44%) | 416 (39.32%) |  |
| No | 229 (31.59%) | 90 (35.43%) | 29 (36.71%) | 138 (39.88%) |  |
| Genetic testing offered | 7 (0.97%) | 24 (9.44%) | 17 (21.52%) | 48 (4.54%) |  |
| Mean estimated heritability in percentages (SD) | 71.67 (21.26) | 73.93 (21.63) | 63.68 (26.63) | 71.61 (21.91) |  |

Note: *it was allowed to choose more than one reason

sTable 3. Comparative Analyses between Dropout and Non-Dropout Participants for Demographic and Dependent Variables.

|  |  | Dropout | Non-Dropout | Statistical test |
| --- | --- | --- | --- | --- |
| **Demographics** | | | | |
| Highest achieved education* | Low | 63 | 68 | X^2^ = 11.83, df = 4, *p* = .003 |
|  | Middle | 142 | 143 |  |
|  | High | 152 | 254 |  |
|  | Other | 3 | 1 |  |
|  | No education | 4 | 2 |  |
| Biological sex** | Male | 398 | 317 | X^2^ = 13.00, df = 1, *p* < .001*** |
|  | Female | 309 | 365 |  |
|  | Other/Unclear | 5 | 4 |  |
| Age | Mean | 32.35 Years | 43.67 Years | T = -12.3, df = 1391.4, *p* < .001*** |
| Age at diagnosis | Mean | 25.57 Years | 33.22 Years | T = -7.39, df = 1216, *p* < .001*** |
| **Dependent variables** | | | | |
| Importance heritability | Very important | 554 | 526 | X^2^ = 1.80, df = 2, *p* = .407 |
|  | A little important | 115 | 126 |  |
|  | Not important | 43 | 34 |  |
| Wants to know more | Yes | 472 | 437 | X^2^ = .92, df = 1, *p* = .338 |
|  | No | 240 | 249 |  |

*‘Other’ and ‘No Education’ were not taken into chi-square test due to their low counts
** ‘Other/Unclear’ was not taken into chi-square test due to its low count
*** Statistically significant

sTable 4. Cross-Sectional Analyses on the Change in Perspectives on Autism Genetics Research over 5-Year Period (2018 – 2023) in the Total Sample and per Stakeholder Group.

|  | Adults | | |  | Parents | | |  | Representatives | | |  | Total sample | | |  |  |
| --- | --- | --- | --- | --- | --- | --- | --- | --- | --- | --- | --- | --- | --- | --- | --- | --- | --- |
|  | X^2^ | Df | *p* | N | X^2^ | Df | *p* | N | X^2^ | Df | *p* | N | X^2^ | Df | *p* | *N* |  |
| Heritability important | 1.92 | 2 | .383 | 1760 | 7.61 | 2 | *.022* | 449 | 1.69 | 2 | *.431* | 126 | 3.71 | 2 | *.157* | *2328* | |
|  |  |  |  |  |  |  |  |  |  |  |  |  |  |  |  |  | |
| Reasons |  |  |  |  |  |  |  |  |  |  |  |  |  |  |  |  | |
| Knowledge | 0.84 | 1 | .360 | 1657 | 0 | 1 | *1* | 431 | 1.85 | 1 | *.173* | 119 | 0.08 | 1 | *.777* | *2200* | |
| Acceptance | 3.28 | 1 | .070 | 1657 | 3.41 | 1 | *.065* | 431 | 0 | 1 | *1* | 119 | 12.19 | 1 | *<.001** | *2200* | |
| Diagnosis | 1.81 | 1 | .179 | 1657 | 0.27 | 1 | *.606* | 431 | 0.796 | 1 | *.372* | 119 | 2.75 | 1 | *.097* | *2200* | |
| Co-occurrence | 0.05 | 1 | .830 | 1657 | 0.12 | 1 | *.730* | 431 | 0 | 1 | *1* | 119 | 0.155 | 1 | *.693* | *2200* | |
| Help | 2.86 | 1 | .091 | 1657 | 2.58 | 1 | *.108* | 431 | 0.34 | 1 | *.561* | 119 | 4.21 | 1 | *.040* | *2200* | |
| Family planning | 1.63 | 1 | .202 | 1657 | 0.14 | 1 | *.705* | 431 | 0.150 | 1 | *.699* | 119 | 1.30 | 1 | *.254* | *2200* | |
| Want to know more | 0.64 | 1 | .424 | 1760 | .50 | 1 | *.480* | 449 | 1.83 | 1 | *.176* | 126 | 0 | 1 | *1* | *2328* | |
|  |  |  |  |  |  |  |  |  |  |  |  |  |  |  |  |  | |

*Significant after Bonferroni correction.

sTable 5. Longitudinal Analyses on the Change in Perspectives on Autism Genetics Research over 5-Year Period (2018 – 2023) in the Total Sample and per Stakeholder Group.

|  | Adults | | |  | Parents | | |  | Representatives | | |  | Total sample | | |  |  |
| --- | --- | --- | --- | --- | --- | --- | --- | --- | --- | --- | --- | --- | --- | --- | --- | --- | --- |
|  | X^2^ | Df | *p* | N | X^2^ | Df | *p* | N | X^2^ | Df | *p* | N | X^2^ | Df | *p* | *N* |  |
| Heritability important | 2.014 | 3 | *.570* | 579 | 3.00 | 3 | *.392* | 56 | NA | NA | *NA* | 51 | 2.593 | 3 | *.459* | *686* | |
|  |  |  |  |  |  |  |  |  |  |  |  |  |  |  |  |  | |
| Reasons |  |  |  |  |  |  |  |  |  |  |  |  |  |  |  |  | |
| Knowledge | 0.680 | 1 | *.410* | 526 | .563 | 1 | *.453* | 52 | 1.25 | 1 | *.264* | 46 | 0.789 | 1 | *.374* | *624* | |
| Acceptance | 2.312 | 1 | *.128* | 526 | 0 | 1 | *1* | 52 | 0 | 1 | *1* | 46 | 2.227 | 1 | *.136* | *624* | |
| Diagnosis | 0.807 | 1 | *.369* | 526 | 0.962 | 1 | *.327* | 52 | 0 | 1 | *1* | 46 | 1.751 | 1 | *.186* | *624* | |
| Co-occurrence | 1.340 | 1 | *.247* | 526 | 0 | 1 | *1* | 52 | 0 | 1 | *1* | 46 | 1.415 | 1 | *.234* | *624* | |
| Help | 0.335 | 1 | *.563* | 526 | 0 | 1 | *1* | 52 | 1.067 | 1 | *.302* | 46 | 0.754 | 1 | *.385* | *624* | |
| Family planning | 4.198 | 1 | *.040* | 526 | 0 | 1 | *1* | 52 | 0 | 1 | *1* | 46 | 3.406 | 1 | *.065* | *624* | |
| Want to know more | 16.144 | 1 | *<.001* | 579 | 1.45 | 1 | *0.228* | 56 | .063 | 1 | *.803* | 51 | 15.602 | 1 | *<.001** | *686* | |
|  |  |  |  |  |  |  |  |  |  |  |  |  |  |  |  |  | |

*Significant after Bonferroni correction.
